# Efficacy of light based detection systems for early detection of oral cancer and oral potentially malignant disorders: Systematic review

**DOI:** 10.4317/medoral.21104

**Published:** 2016-03-06

**Authors:** Ravleen Nagi, Yashoda-Bhoomi Reddy-Kantharaj, Nagaraju Rakesh, Sujatha Janardhan-Reddy, Shashikant Sahu

**Affiliations:** 1 Senior Lecturer. Department of Oral Medicine and Radiology, New Horizon Dental College and Research Institute, Sakri, Bilaspur, Chattisgarh; 2 Senior Professor. Department of Oral Medicine and Radiology, Faculty of Dental Sciences, MS Ramaiah University of Applied Sciences, MSRIT Post, Mathikere, Bangalore, Karnataka, India; 3 Reader. Department of Oral Medicine and Radiology, Faculty of Dental Sciences, MS Ramaiah University of Applied Sciences, MSRIT Post, Mathikere, Bangalore, Karnataka, India; 4 Professor and Head. Department of Oral Medicine and Radiology, Faculty of Dental Sciences, MS Ramaiah University of Applied Sciences, MSRIT Post, Mathikere, Bangalore, Karnataka, India; 5 Consultant. Plastic Surgeon, Burn and Trauma Centre, Bilaspur, Chattisgarh

## Abstract

**Background:**

Earlier detection of oral squamous cell carcinoma (OSCC) and oral potentially malignant disorders (OPMD) is essential for dental professionals to improve patient survival rates. The aim of this systematic review is to to evaluate the effectiveness of devices that utilise the principles of chemiluminescence and tissue autofluorescence as adjuncts in the detection of OSCC and OPMD.

**Material and Methods:**

The electronic retrieval systems and databases searched for relevant articles were PubMed [MEDLINE] and Science direct. The search was for limited articles published in English or with an English abstract and articles published during the period from January 2005 to April 2014. Clinical trials utilized ViziLite, Microlux TM/DL and Visual Enhanced Light scope (VELscope) for early detection of OPMD and OSCC.

**Results:**

Twenty primary studies published satisfied our criteria for selection - 10 utilised chemiluminescence and 10 tissue autofluorescence. Senstivity of Vizilite for detecting OSCC nad OPMD ranged from 77.1 % to 100% and specificity was low that ranged from 0% to 27.8%.Most have shown that chemiluminescence increases the brightness and margins of oral mucosal white lesions and thus assist in identification of mucosal lesions not considered under Conventional visual examination. However, it preferentially detects leukoplakia and may fail to spot red patches. Clinical trials demonstrated that sensitivity of VELscope in detecting malignancy and OPMD ranged from 22 % to 100 % and specificity ranged from 16 % to 100%. Most studies concluded that VELscope can help the experienced clinician to find oral precursor malignant lesions. But it couldnot differentiate between dysplasia and benign inflammatory conditions.

**Conclusions:**

Both devices are simple, non-invasive test of the oral mucosa but are suited for clinicians with sufficient experience and training. More clinical trials in future should be conducted to establish optical imaging as an efficacious adjunct tool in early diagnosis of OSCC and OPMD.

**Key words:**Oral cancer, early diagnosis, potentially malignant disorders, chemiluminescence, tissue autofluorescence, VELscope, ViziLite plus.

## Introduction

Oral malignancies are one of the most common cancers around the world and ranks sixth to eighth among cancers in various studies. These cancers are major economic and clinical burden for the health care around the world (1). In India, oral cancer represents a major health problem accounting for upto 40 % of all cancers, and is most common cancer in males and third most common cancer in females. It often arises from Oral potential malignant disorders (OPMDs) such as erythroplakia, leukoplakia and oral Lichen planus (2). Leukoplakia is the most common OPMD and its worldwide prevalence is approximately 2.6% (3).

Risk factors for oral cancer are well established and include tobacco and alcohol use (4). Despite the established risk factors and advances in treatment, the 5-year survival for oral squamous cell carcinoma (OSCC) associated with tobacco and alcohol use has remained consistently poor for the last forty years (5). Prognosis is further complicated by the high rate of second primary tumours in these patients, which is thought to be the result of ‘field cancerisation’ in the upper aerodigestive tract (6).

Early detection of neoplastic changes in the oral cavity is the best method to improve patient survival rates (7). The current method of oral cancer diagnosis, visual examination of the oral cavity, relies heavily on clinical expertise in recognizing early neoplastic changes. However, discerning premalignant and early malignant lesions from common benign inflammatory conditions by visual examination is difficult, even for experienced practitioners (8). Many techniques to date have been reviewed so far e.g. vital staining procedure (Toulidine Blue and Lugols iodine), Brush Biopsy (Oral CDx Brush),micronuclei anlaysis, DNA ploidy but have certain limitations (2). Light-based techniques, including chemiluminescence and autofluorescent imaging, work on the assumption that neoplastic and pre-neoplastic tissues that have undergone abnormal metabolic or structural changes have different absorbance and reflectance properties when exposed to specific wavelengths of light. In the last decade, light-based technology has been adapted and marketed for use in the oral cavity (chemiluminescence: ViziLite, ViziLite Plus, MicroLuxTM/DL; autofluorescence: VELscope (Visual Enhanced Light scope) (9). The objective of this systematic review is to evaluate the literature investigating the effectiveness of chemiluminescence and autofluorescent imaging devices as aids in the detection of OSCC and OPMDs and encouraging dental professionals to use these light based detection devices in clinical practice.

## Material and Methods

A systematic review of the scientific literature was done in preparation of manuscript. The electronic retrieval systems and databases searched for relevant articles were PUBMED [MEDLINE] and SCIENCE DIRECT. Database of indexed journals were searched for keywords such as Oral cancer, early diagnosis, potentially malignant disorders, chemiluminescence; tissue autofluorescence, VELscope; ViziLite Plus. The inclusion criteria were the use of light based techniques for early diagnosis of OSCC or OPMD, publications reporting primary studies and publications written in English. The exclusion criteria were case reports, reviews and studies in other languages.

## Results

For the use of chemiluminescence aids (ViziLite, ViziLite plus and MicroluxTM DL) in the detection of OPMD and OSCC ten studies satisfied our inclusion and exclusion criteria. These studies were conducted in clinics of countries such as Malaysia (10), Australia (11,12), USA (13), India (14-16) and UK (17). Most studies used ViziLite to detect OPMD and OSCC but one study used Microlux TM/DL (12).  Table 1 1 and  Table 1 continue, illustrates the clinical trials conducted in literature establishing the role of chemiluminescence in detection of OPMD and OSCC.

**Table 1:**
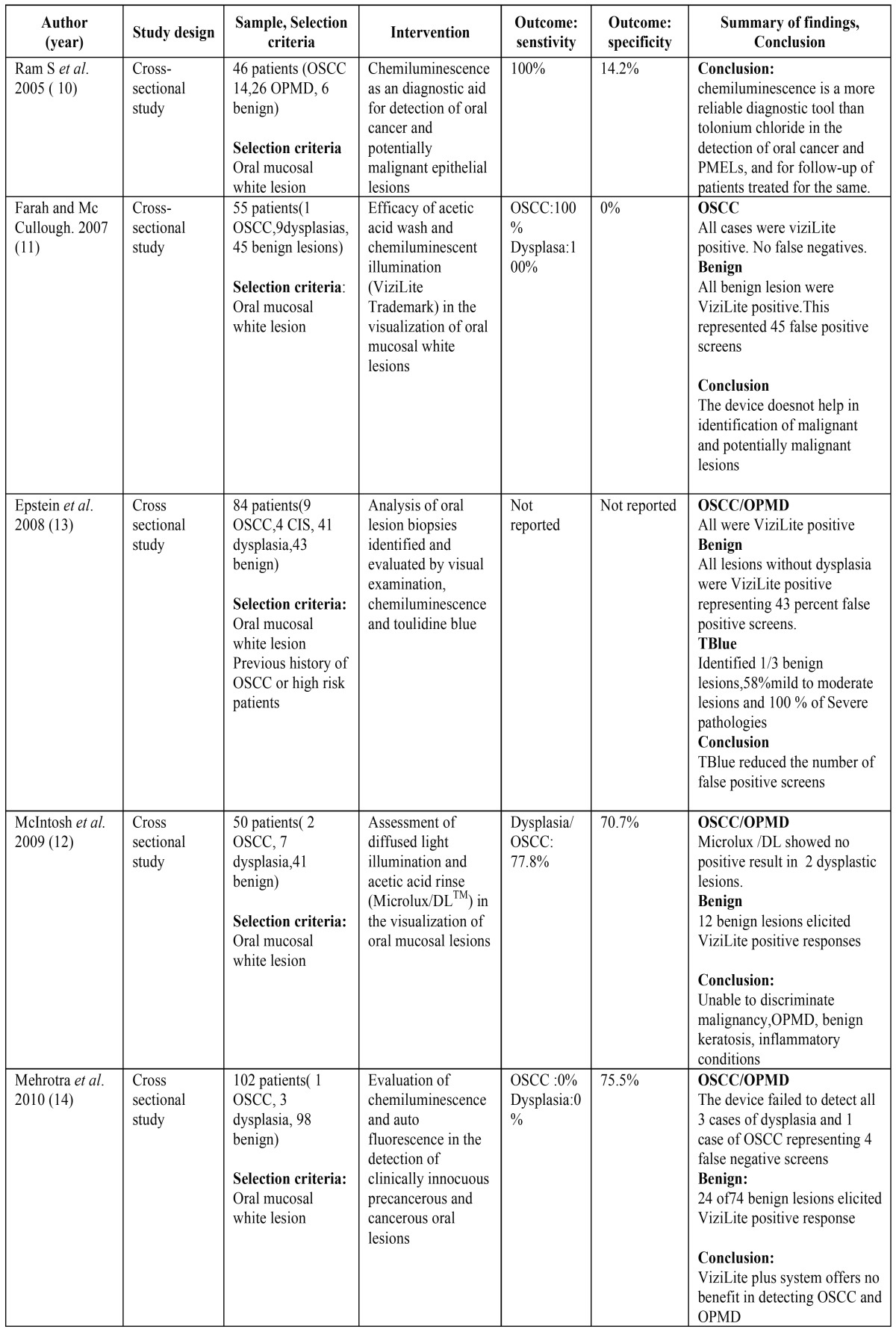
Summarizes the clinical trials to evaluate the efficacy of chemiluminescence in detection of oral cancer and oral potentially malignant disorders.

**Table 1 continue:**
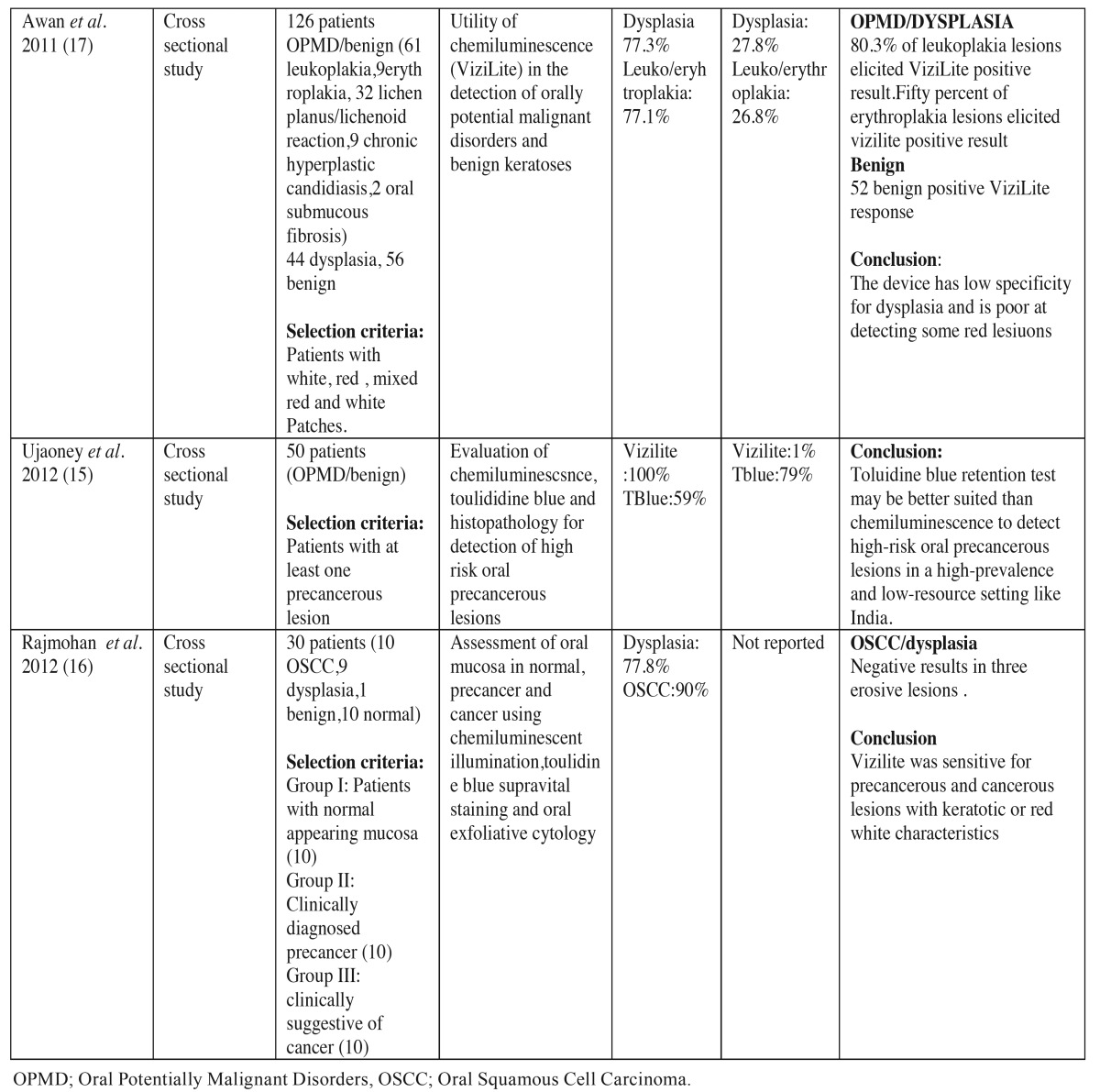
Summarizes the clinical trials to evaluate the efficacy of chemiluminescence in detection of oral cancer and oral potentially malignant disorders.

Most studies were cross sectional studies and several parameters were considered for correct evaluation. The sensitivity of a test, is the proportion of people who test positive for a specific disease among a group of people who have the disease. Specificity is the proportion of people who test negative for a specific disease among a group of people who do not have the disease. False positive is an erroneously positive test or screening result. False negative is an erroneously negative test or screening result.

Senstivity of Vizilite for detecting OSCC and OPMD ranged from 7.1 % to 100% and specificity was low that ranged from 0% to 27.8%. In a study by Ram et al. the sensitivity of vizilite was 100% and specificity was low 14.2 % (10). Ujaoney et al. found toulidine was better suited than chemiluminescence for detecting high risk patients (15). McIntosh *et al.* used Microlux DL in his study with sensitivity of 77.8 % and specificity of 70.7%. in detecting dysplasia and OSCC but Microlux TM/DL couldnot discriminate between malignancy, OPMD, benign keratosis and inflammatory conditions (12).

For the use of VELscope in detection of OSCC and OPMD ten studies in literature satisfied our inclusion and exclusion criteria. These studies were mainly crosssectional and were carried out in clinics of countries such as UK (18), Canada (19), Germany (20-22), Italy (23), USA (24,25), Poland (26) and India (14). Clinical trials demonstrated that sensitivity of VELscope in detecting malignancy and OPMD ranged from 22 % to 100 % and specificity ranged from 16 % to 100%.Most studies concluded that VELscope can help the experienced clinician to find oral precursor malignant lesions (20,22,25).  Table 2 and  Table 2 continue , summarizes clinical trials conducted in literature to test the efficacy of VELscope in early diagnosis of high risk patients and OPMD.

**Table 2:**
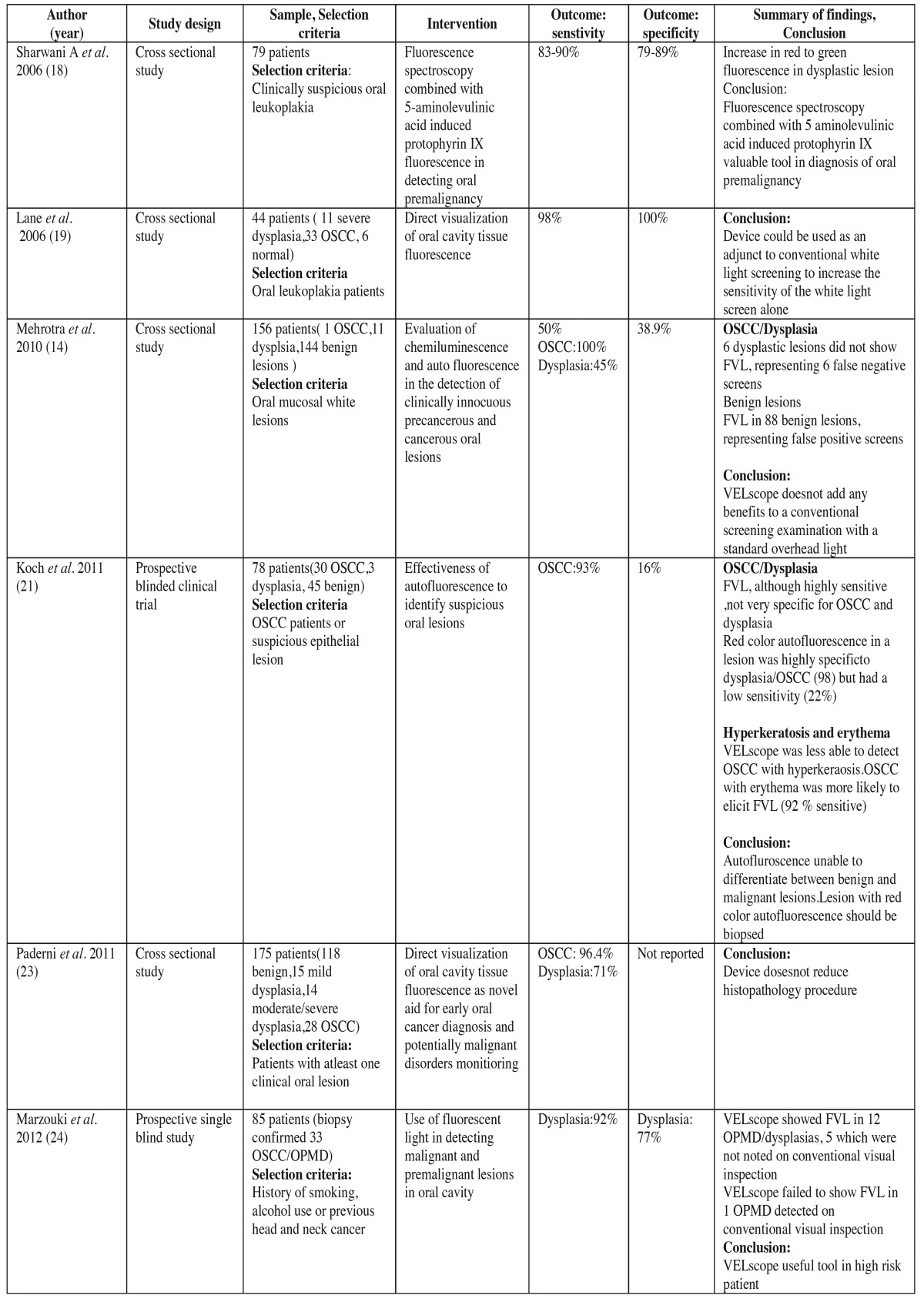
Summarizes clinical trials to evaluate the efficacy of autofluorescence imaging ( VELscope) in detecting oral cancer and oral potentially malignant disorders.

**Table 2 continue:**
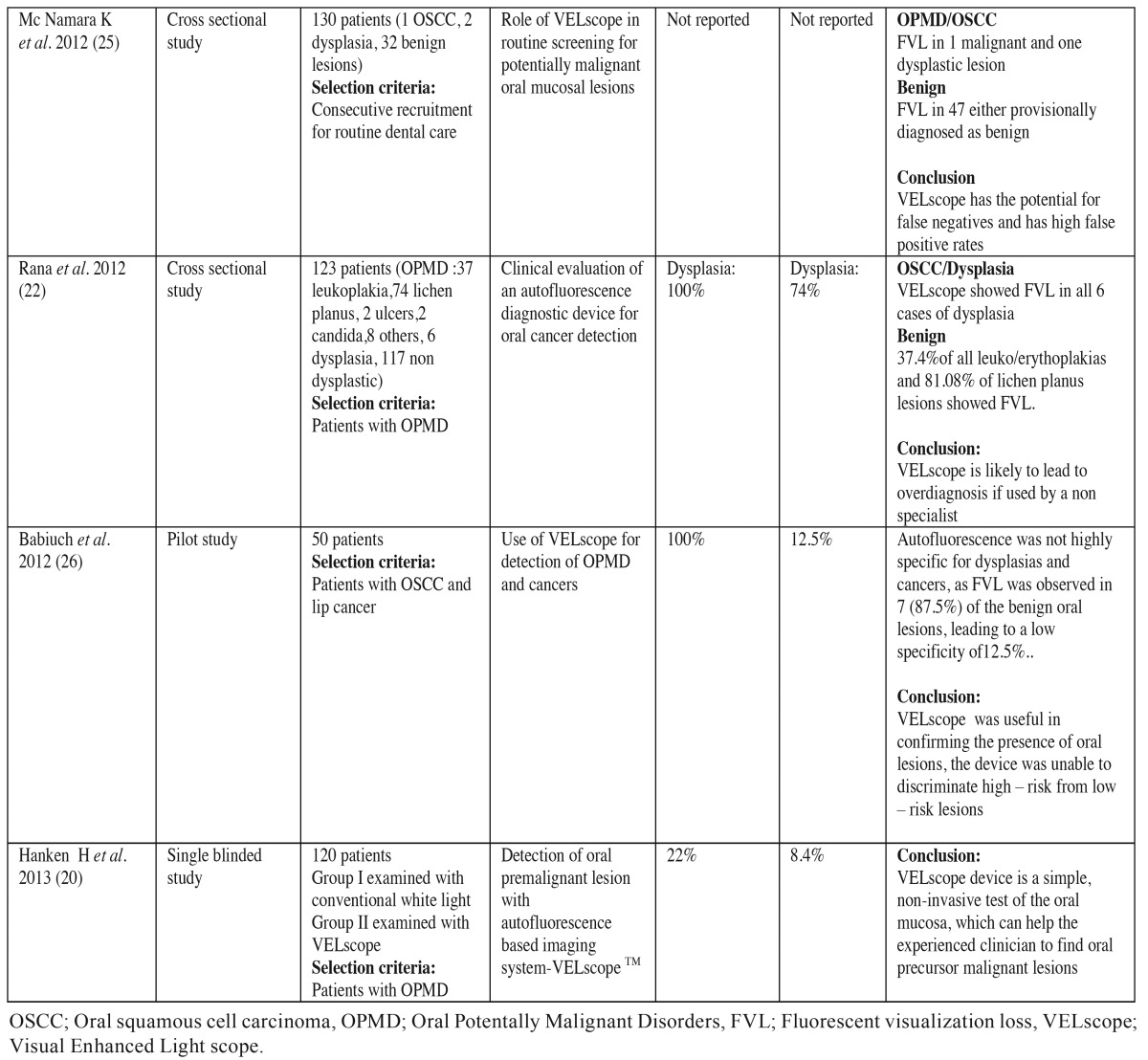
Summarizes clinical trials to evaluate the efficacy of autofluorescence imaging ( VELscope) in detecting oral cancer and oral potentially malignant disorders.

## Discussion

- Chemiluminescence

Chemiluminescence involves emission of light from a chemical reaction between hydrogen peroxide and acetylsalicylic acid inside a capsule light stick. This reaction emits blue/white light (430-580 nm) whose principle is based on the reflective properties of tissues that present cellular alterations such as a higher nuclear/cytoplasmatic rate. The acetowhite lesion is more defined, whereas the normal tissue is dark. Chemiluminescence was first applied for the detection of dysplasia in the cervix. The test has recently been adapted and proposed for oral mucosal examination based on the hypothesis that oral mucosal tissues may exhibit features similar to the cervical epithelium when subjected to chemiluminescence (27). One of the components of chemiluminescent examination is acetic acid pre-rinse. It is mainly done to remove the debris and glycoprotein layer for enhanced penetration and reflection of light. But acetic acid is also known to cause cellular dehydration and protein coagulation that reduces the transparency of the epithelium.This could be one of the reasons for the aceto-white appearance of the white lesions (28).

Various studies have been done in literature to evaluate efficacy of Vizilite, some have shown conflicting results. Most have shown that chemiluminescence increases the brightness and margins of oral mucosal white lesions and thus assist in identification of mucosal lesions not detected under Conventional visual examination (COE). Ram *et al.* found that ViziLite was 100 % sensitive with a low specificity of 12.5 % for detection of OPMD and OSCC (10). Rajmohan *et al.* assessed oral mucosa in normal, precancer and cancer patients using ViziLite and it was found 77.8 % sensitive for detecting dysplasia and 90 % sensitive for detecting OSCC (16). In a study by Awan *et al., *the majority of mucosal disorders were positive (aceto-white) for chemiluminescence (75.4%). ViziLite was useful in enhancing the visibility and sharpness of majority of the oral leukoplakia, making the clinically evident lesions more prominent and distinct from surrounding oral mucosa. Fifty percent erythroplakia lesions were ViziLite positive (17). 

There are many limitations associated with the use of Vizilite: Examination needs a dark environment, high cost, no permanent record unless photographed,low specificity for dysplasia, contributing to high referral rate and over-treatment, unable to detect some red lesions, acetic acid pre-rinse increases salivary flow that interferes with mucosal surface reflectance, inability to objectively measure the visualization results. This visualization adjunct gives information only about the horizontal extent of the lesion (one dimension). The depth of the lesion which is more important in predicting the malignant behavior cannot be assessed through this modality (11).

Various studies proved that Vizilite is not a reliable tool to detect early premalignancy. In a study by Awan *et al.* majority of leukoplakias (80.3%) showed acetowhitening in contrast to only half of the erythroplakias. This clearly demonstrates the ability of the ViziLite to detect leukoplakias (white patches) more accurately and also indicates the inability of ViziLite to detect or enhance some red patches (erythroplakias). The ability of the ViziLite to detect dysplastic lesions has been greatly undermined by failure of the device to distinguish dysplastic from non-dysplastic lesions (sensitivity - 77.3%, specificity - 27.8%) (17). Mehrotra *et al.* found that Vizilite was not sensitive (0%) in detecting dysplasia and OSCC and has no benefit in detecting OSCC and OPMD (14). Ujaoney *et al.* used chemiluminescence and Toulidine Blue for detecting of high risk oral precancerous lesions and Toulidine blue was found to be better diagnostic test than chemiluminesecence (15).

- Tissue autofluorescence

The autofluorescence of tissue and its potential use in cancer detection were described first in 1924. It is a phenomenon where by an extrinsic light source is used to excite endogenous fluorophores such as certain amino acids, metabolic products, and structural proteins. Within the oral mucosa, the most relevant fluorophores are nicotinamide adenine dinucleotide (NADH) and flavin adenine dinucleotide (FAD) in the epithelium and collagen cross-links in the stroma. The fluorophores absorb photons from the exogenous light source and emit lower energy photons which present clinically as fluorescence (23). Each fluorophore is associated with specific excitation and emission wavelengths. When irradiated with wavelengths between 375 and 440 nm,the fluorochromes show fluorescence in the green spectral range and normal, unaltered mucosa emits a pale green autofluorescence when viewed through a selective, narrowband filter. A proper filtration is crucial, due to the intense light used for excitation of the fluorochromes. Without a proper filtration, it would be impossible to visualize the pale and narrow autofluorescence signal. However, dysplastic tissues lose fluorescence emission power due to a disruption in the distribution of the fluorochromes and appear darker in colour in comparison to the surrounding healthy tissue (29).

A number of methods based on the principles of tissue fluorescence have been described for use in the oral cavity, including exogenous fluorescence, autofluorescent spectroscopy and autofluorescent imaging. Both exogenous fluorescence and autofluorescent spectroscopy due to practical purposes are unlikely to be applied as screening aids. In exogenous fluorescence, there is a delay before the fluorophore reaches an adequate concentration and the fluorophore also causes temporary photosensitisation to the subject, which may be deemed unacceptable to the individual. In autofluorescence spectroscopy, small optical fibres are used to expose the oral mucosa to different wavelengths of light and it is not possible to screen the entire oral cavity, therefore limiting its application. For these reasons, this review will focus on the use of autofluorescent imaging.

VELscope utilises blue light excitation between 400 and 460 nm wavelength to enhance oral mucosal abnormalities by direct tissue autofluorescence. At these excitation wavelengths, normal oral mucosa is associated with a pale green fluorescence when viewed through a filter, whereas abnormal tissue is associated with a loss of autofluorescence and appears dark. Neoplastic tissues are expected to cause fluorescent visualisation loss (FVL) and thus appear as a dark area (30).

Several studies have investigated the effectiveness of the VELscope system as an adjunct to visual examination for 1) improving the distinction between normal and abnormal tissues (both benign and malignant changes) 2) differentiating between benign and dysplastic/malignant changes 3) and identifying dysplastic/malignant lesions that are visible to naked eye under white light. Whether it can distinguish between dysplasia and benign inflammatory lesions is questioned. Benign inflammatory conditions can result in an increased blood supply to a lesion. The increased haemoglobin content (chromophores) may absorb light and cause FVL mimicking neoplasia (24,25).

Hanken H *et al.* examined 120 patients with suspicious oral lesions and found VELscope has a higher sensitivity (22.0%), and a lower specificity (8.4%). Also it is more promising than COE in detecting precursor oral malignant lesions (20). Koch *et al.* in his study showed a higher sensitivity (97%) and specificity of (95.8%) of VELscope to diagnose OSCC. The positive predictive value (PPV) was calculated was 41% and negative predictive value (NPV) was 75-80% (21). Rana *et al.* in his study showed that using the VELscope leads to higher sensitivity (100% vs. 17%), but a lower specificity (74% vs. 97%) as compared to COE. The major lack of the study was the large number of false-positive test results (22). In another study McNamara *et al.* concluded that COE is more valid than autofluorescence examination with VELscope in routine screening for OPMD (25). They believed that careful, systematic visual and tactile examination of the entire oral cavity on a regular basis remains the gold standard for early detection of OPMD. Babiuch *et al.* found in his study that autofluorescence was not highly specific for dysplasias and cancers, as FVL was observed in 7 (87.5%) of the benign oral lesions, leading to low specificity of 12.5 %. But this device was unable to discriminate high risk from low risk lesions (26).

## Conclusions

Detection of OPMDs before they advance to OSCC is necessary to improve survival rates for oral cancer. Evidence indicates that COE is a poor discriminator of oral mucosal lesions, and this has led to the development of several adjunctive visualisation aids. Both devices are simple, non-invasive tests of the oral mucosa, which can help the experienced clinician to find oral precursor malignant lesions and the correct location for taking biopsies within the altered mucosa. But in the literature, both techniques have limited ability to discriminate the high-risk lesions and have limitations which limit their use. In any case, conventional visual inspection under normal incandescent light, followed by biopsy of suspicious lesions, will remain the gold standard for the immediate future. Future approaches to optical imaging could involve real time quantitative evaluation to determine a diagnosis for oral mucosal lesions rather than simply highlighting the presence of abnormalities, thus, making the possibility of “optical biopsy” a clinical reality.
